# Childhood maltreatment and the structural development of hippocampus across childhood and adolescence

**DOI:** 10.1017/S0033291724001636

**Published:** 2024-12

**Authors:** Victoria Fogaça Doretto, Ana Beatriz Ravagnani Salto, Sandra Schivoletto, Andre Zugman, Melaine Cristina Oliveira, Marcelo Brañas, Marcos Croci, Lucas Toshio Ito, Marcos Santoro, Andrea P. Jackowski, Rodrigo A. Bressan, Luis Augusto Rohde, Giovanni Salum, Eurípedes Constantino Miguel, Pedro Mario Pan

**Affiliations:** 1 Department of Psychiatry Hospital das Clínicas, Faculdade de Medicina, Universidade de São Paulo, São Paulo, Brazil; 2 National Institute of Developmental Psychiatry for Children and Adolescents, São Paulo, Brazil; 3 Department of Psychiatry, Laboratório Interdisciplinar Neurociências Clínicas (LiNC), Universidade Federal de São Paulo, São Paulo, Brazil; 4 Department of Education, Information and Communications Technology (ICT) and Learning, Østfold University College, Halden, Norway; 5 Attention-Deficit/Hyperactivity Disorder and Developmental Psychiatry Programs, Hospital de Clínicas de Porto Alegre, Universidade Federal Do Rio Grande Do Sul, Porto Alegre, Brazil; 6 Department of Psychiatry, Faculdade de Medicina, Universidade Federal do Rio Grande do Sul, Porto Alegre, Brazil

**Keywords:** brain development, childhood maltreatment, hippocampus, longitudinal, MRI

## Abstract

**Background:**

Prior studies suggest that childhood maltreatment is associated with altered hippocampal volume. However, longitudinal studies are currently scarce, making it difficult to determine how alterations in hippocampal volume evolve over time. The current study examined the relationship between childhood maltreatment and hippocampal volumetric development across childhood and adolescence in a community sample.

**Methods:**

In this longitudinal study, a community sample of 795 participants underwent brain magnetic resonance imaging (MRI) in three waves spanning ages 6–21 years. Childhood maltreatment was assessed using parent-report and children´s self-report at baseline (6–12 years old). Mixed models were used to examine the relationship between childhood maltreatment and hippocampal volume across time.

**Results:**

The quadratic term of age was significantly associated with both right and left hippocampal volume development. High exposure to childhood maltreatment was associated with reduced offset of right hippocampal volume and persistent reduced volume throughout adolescence.

Critically, the relationship between childhood maltreatment and reduced right hippocampal volume remained significant after adjusting for the presence of any depressive disorder during late childhood and adolescence and hippocampal volume polygenic risk scores. Time-by-CM and Sex-by-CM interactions were not statistically significant.

**Conclusions:**

The present study showed that childhood maltreatment is associated with persistent reduction of hippocampal volume in children and adolescents, even after adjusting for the presence of major depressive disorder and genetic determinants of hippocampal structure.

## Introduction

Childhood maltreatment (CM) is a serious public health issue that adversely affects victims, their families, and society as a whole (Gilbert et al., 2009). Exposure to CM constitutes a major risk factor for psychiatric disorders, such as major depressive disorder (MDD) (Cohen, Brown, & Smaile, 2001) and other negative psychological outcomes (Schäfer et al., 2023). Though the specific neural mechanisms underlying the relationship between CM and MDD are not completely understood, emerging evidence from adult samples suggests that both are associated with structural disruptions in subcortical brain regions, particularly the hippocampus (Hanson et al., 2015). Here, we sought to identify CM-related hippocampal abnormalities across neurodevelopment and their relationship with child- and adolescent-onset MDD.

The hippocampus is a limbic structure involved in cognitive processes (e.g. associative learning, memory formation and consolidation) (Euston, Gruber, & McNaughton, 2012), emotional regulation, and the stress response (Smith & Vale, 2006). Previous large-scale studies indicate that typical hippocampal volumetric development peaks during adolescence (Tamnes, Bos, van de Kamp, Peters, & Crone, 2018). Therefore, this subcortical region may be specifically impacted by early life exposure to trauma. Studies in children and adolescents have found conflicting results on the association between structural hippocampal development and CM. For example, cross-sectional studies have shown either decreased hippocampal volume related to CM (Hanson et al., 2015; Herzog et al., 2020), no significant differences (De Brito et al., 2013; Gold et al., 2016) or increased volume in subjects exposed to CM (Herzog et al., 2020; Tupler & De Bellis, 2006)

To date, few longitudinal studies examined the relationship between hippocampal structural development and CM. Paquola et al. (2017) studied 123 adolescents exposed to CM (obtaining follow-up scans for approximately half of these participants) and reported attenuated volumetric growth of the hippocampus in the CM group compared to controls (Paquola et al., 2017). Contrastingly, a three-wave longitudinal study including 166 adolescents found higher hippocampal volume at baseline and at first follow-up visit among participants with a history of CM. By late adolescence, however, this group showed a ‘catch-up’ effect on their hippocampal volumetric development (Whittle et al., 2017). While these longitudinal studies provide a critical starting point, they did not adequately account for the presence of common psychopathology, such as MDD (Paquola et al., 2017; Whittle et al., 2013, 2017). This is key given that the complex interplay between CM and hippocampal volumetric development may be impacted by MDD via common pathophysiological pathways (e.g. HPA axis, brain growth factors (Barch et al., 2019; Malhi & Mann, 2018).

Another important gap in previous literature on this topic is the scarcity of studies conducted in Low-and-Middle Income Countries (LaMIC), where most of the world's youth population lives (Nations, 2001). This gap aligns with findings presented by Battel et al. (2021), who highlight a profound underrepresentation of LaMIC cohorts in neuroimaging research (Battel et al., 2021). The need for a more inclusive research database is further substantiated by studies suggesting an elevated risk of CM among children residing in LaMIC (Viola et al., 2016).

Here, taking advantage of longitudinal neuroimaging data from an ongoing developmental cohort, we aimed to investigate the relationship between CM and hippocampal volume across childhood and adolescence. CM was assessed at baseline, when participants were 9 years-old (on average), and then additional imaging data were collected at 3- and 6-year follow-up visits. We hypothesized that CM would be associated with altered baseline hippocampal volume and altered hippocampal growth trajectories. Finally, we scrutinized the CM-Hippocampal volumetric link by performing analyses including mental disorders, with a specific focus on MDD (Schmaal et al., 2016), and adjusting for genetic determinants of hippocampal structure (Hibar et al., 2017).

## Methods

### Participants

#### Screening phase (Year 2009)

This study was conducted using data from the Brazilian High-Risk Cohort Study (BHRC). In 2009, parents of 6–12-years-old children from 57 schools in the cities of São Paulo and Porto Alegre were invited to participate in a cohort study. On the school registry day, biological parents answered the Family History Screen (FHS) (Weissman et al., 2000) for 8012 families, representing 9937 children. From this pool, two subgroups were recruited – one randomly selected (*n* = 958), aimed to mirror community demographics, and one high-risk sample based on their risk for mental disorders (*n* = 1554) according to symptoms reported in FHS, resulting in a total of 2511 participants. Only one child per family was included. All parents/guardians signed informed consent and children provided verbal assent. The ethics committees of all three universities involved in the cohort approved the project. For a detailed description of BHRCS sampling procedure, see Salum *et al*. (2015) (Salum et al., 2015).

#### Baseline assessment (Year 2010–11), 3- (Year 2013–2015) and 6-year follow-up (Year 2017–2019)

After the screening phase, parents were invited to participate in the baseline assessment phase. This phase was performed in multiple visits and included a parent interview and three child evaluation sessions. The parent interview consisted of a household lay interview conducted with a biological parent (the mother in 94.5% of cases) and included a detailed evaluation of general risk factors for mental conditions and a structured psychiatric interview using the Developmental and Well-Being Assessment (DAWBA – Brazilian Portuguese version) (Goodman, Ford, Richards, Gatward, & Meltzer, 2000; Goodman, Heiervang, Collishaw, & Goodman, 2011). The child evaluation was administered by trained psychologists and speech therapists. From the total phenotypic baseline sample (*n* = 2511), the project was granted to perform brain MRI scans for a subsample of 750 children. A total of 741 participants underwent structural MRI scan at baseline. All subjects who completed the baseline brain MRI evaluation were invited for follow-up scans at the 3- and 6-year follow-up phases. However, not all individuals in the original neuroimaging sample were scanned at follow-up visits due to medical restrictions (mostly dental braces) or refusal to attend the MRI session. Therefore, 97 additional individuals from the cohort without baseline MRI scans were invited to a brain scan session at the 3-year follow-up wave. Six years after the baseline evaluation, individuals with previous scans at Baseline and/or W1 were again invited to participate. A total of 547 and 414 subjects successfully completed the 3- and 6-year follow-up MRI brain scans, respectively. The final sample consisted of a total of 795 participants. Please see online Supplementary Fig. S1 in the data supplement for a flowchart with detailed information.

### Childhood maltreatment assessment

In the context of this large cohort study, questions about lifetime exposure to CM were included to investigate four categories of maltreatment: (a) physical abuse (infliction of bodily injury by non-accidental means); (b) sexual abuse (sexual contact or attempted contact for purposes of sexual gratification or financial gain); (c) emotional maltreatment (pervasive and extreme thwarting of a child's basic emotional needs); and (d) neglect (failure to provide minimum care and/or lack of supervision) (Barnett, Manly, & Cicchetti, 1993). At baseline, the CM section consisted of seven questions, four responded by biological parents and three by children themselves, in distinct interviews and locations (Parents – household lay interview, Children – school-based psychologist interview). Responses to these seven questions were rated on a 4-point scale: 0, never; 1, one or two times; 2, sometimes; 3, frequently). Please see Supplemental Methods for a detailed description of the CM items and online Supplemental Table S1 for endorsement rates of every item at cohort baseline.

Similar questions have been frequently used in survey research (Arseneault et al., 2011) and they are close to the constructs evaluated by other instruments that assess CM more comprehensively, such as the Childhood Trauma Questionnaire (Bernstein et al., 2003; Bernstein, Ahluvalia, Pogge, & Handelsman, 1997). Only baseline CM data was included in the present analyses, since our aim was to investigate the effect of trauma that occurred during childhood. The item on sexual abuse was asked only of parents following local IRB recommendations.

To integrate data from both child and parent reports and simultaneously create a unified measure of CM, we employed Confirmatory Factor Analysis (CFA) to CM items. We utilized a higher-order model with two lower-order factors (parent- and child-report) and a higher-order factor (CM) that presented the best fit to the data, as described in detail by Salum et al. (2016) (Salum et al., 2016). This analysis generated individual factor scores for the higher-order CM factor based on information from both informants. As previously published, an item-level thresholds of the CFA was used to generate a dichotomous categorical classification of CM exposure (‘low’ *v.* ‘high’ CM exposure) with a value of 1.5 deemed as indicative of high exposure. For parental report, high CM exposure was then defined as: physical abuse and physical neglect rated sometimes or frequently, sexual abuse rated rarely, sometimes or frequently, and emotional abuse rated frequently. High exposure in children's reports was defined as: physical abuse rated sometimes or frequently, physical neglect rated rarely, sometimes or frequently, and emotional abuse rated frequently. Therefore, the ‘low’ variable designation encompasses individuals with minimal or no reported CM on the investigated items.

### Psychiatric assessment

Child psychiatric diagnosis was established using the Developmental and Well-Being Assessment interview (DAWBA). Previous data indicate that self-reports obtained before the age of 11 are relatively unreliable (Schwab-Stone, Fallon, Briggs, & Crowther, 1994). Hence, only parent-reports of their children were assessed at baseline (Goodman et al., 2000). For the follow-up, both parental reports of their children and adolescents' self-report were used. All data from the DAWBA structured items and open-ended questions were reviewed by trained child and adolescent psychiatrists and assessed according to DSM-IV criteria (Goodman et al., 2011). These professionals were trained and closely supervised by a senior child psychiatrist with extensive experience in rating DAWBA interviews. Any disagreements over a specific diagnosis were further discussed by two child psychiatrists until a consensus was achieved.

### Covariates

Sociodemographic data were collected at all time-points and a Brazilian official instrument was used to assess socioeconomic status (The Economic Classification Criterion Brazil) (ABEP, 2010), which includes validated items on the education level of the head of the household and ownership of household assets (TVs, Bathrooms, etc.). This scale score ranges from 0 (poorest) to 46 points (wealthiest).

### Image acquisition and processing

The image acquisition was carried out in two 1.5 T MRI systems (GE Signa HDX and GE Sigma HD – G.E., USA, in the cities of São Paulo and Porto Alegre, Brazil), one for each study site, running identical imaging protocols. T1-weighted scans (three-dimensional fast spoiled gradient sequence) used the following parameters: up to 160 axial slices for whole brain coverage, TR = 10.9 ms, TE = 4.2 ms, thickness = 1.2 mm, flip angle = 15°; matrix size = 256, FOV = 24 cm, and NEX = 1. Imaging acquisitions were repeated whenever participants moved during the procedure to ensure that optimal quality was obtained. At follow-up, scans occurred within the same MRI system at each site (see hippocampal volume distribution by age in online Supplementary Figs S2 and S3).

Neuroimage processing and analyses were performed using an automated atlas-based Bayesian segmentation method, applied in FreeSurfer version 7.1.1. The hippocampus was selected as the region of interest and bilateral hippocampal volume was computed using FreeSurfer automated subcortical segmentation (Fischl et al., 2002). Given the sensitivity of brain MRI signal and segmentation algorithms to artifacts (e.g. head movement, head tilt) (Ducharme et al., 2016; Hedges et al., 2022), we performed visual quality control inspection of extracted volumes and hippocampal segmentation for all outliers, leading to the exclusion of seven observations from four distinct subjects. In addition, we included the Euler number provided by FreeSurfer as a nuisance variable in all statistical models, which is computed as a measure of the topological complexity of the reconstructed cortical surface. Although this measure was developed for the cortical surface algorithm, it is commonly used as a quality control measure to evaluate the accuracy and integrity of FreeSurfer´s brain reconstructions (Rosen et al., 2018). Intracranial total volume was also included in all analyses.

After applying quality control procedures and excluding subjects with missing data on key variables for the present analyses, our final sample comprised *n* = 1525 brain scan data-points (baseline *n* = 717; Wave 1 *n* = 434; Wave 2 *n* = 374) from 795 individual participants (Table 1, online Supplemental Material Fig. S1).

**Table 1.** Demographics and clinical characteristics of participants with childhood maltreatment

	Baseline (*N* = 717)	Wave 1 (*N* = 434)	Wave 2 (*N* = 374)
Site (São Paulo City)	362.0 (50.5%)	232.0 (53.5%)	195.0 (52.1%)
Gender (female)	306.0 (42.7%)	178.0 (41.0%)	160.0 (42.8%)
Age, years, mean (s.d.)	9.9 (1.8)	13.0 (1.8)	17.6 (1.9)
Any mental disorder (*n*, %)	221.0 (30.8%)	157.0 (36.2%)	114.0 (30.5%)
Any depression (*n*, %)	28.0 (3.9%)	49.0 (11.3%)	60.0 (16.0%)
Socioeconomic score, mean (s.d.)	18.2 (4.4)	18.3 (4.1)	24.5 (7.2)
Child maltreatment (parent-report at baseline)	93.0 (13.0%)	61.0 (14.1%)	52.0 (13.9%)
Child maltreatment (self-report at baseline)	93.0 (13.0%)	69.0 (15.9%)	58.0 (15.5%)
Any child maltreatment (parent- or self-report at baseline)	163.0 (22.7%)	111.0 (25.6%)	93.0 (24.9%)

*N*, number; s.d., standard deviation.

*Note:* The total number of included participants was 795.

### Polygenic risk score

Genomic DNA was isolated from saliva (Oragene) using prepIT-L2P reagent (DNAgenotek). Genotyping was performed using the Global Screening Array (Illumina). Single-nucleotide polymorphisms (SNPs) with a minor allele frequency <1%, locus missingness >10%, or Hardy-Weinberg equilibrium significance <0.000001 were excluded, as were individuals with genotype missingness >10% and an estimation of identity by descent >0.12. Genotypic data were available for 795 subjects.

Hippocampal polygenic risk scores (PRS) were calculated with the PRSice V2.3.2 software package (Euesden, Lewis, & O'Reilly, 2015), using the summary statistics of a genome-wide association study (GWAS) of 33 536 individuals (Hibar et al., 2017). The first 10 principal components (PCs) of the genotyping data were entered as covariates in all models including PRS. For the main analyses, a *p* threshold of 0.01 was selected, which revealed 3582 independent SNPs in the target samples. This *p* threshold was highlighted by PRSice V2.3.2 as the most correlated with hippocampal volume in our sample.

### Statistical analyses

Statistical analyses were conducted in Jamovi Version 2.0. (‘*jamovi* (Version 2.0) [Computer Software] Retrieved from https://www.jamovi.org,’ The jamovi project, 2021) and R version 4.1.2 (R Core Team, 2020; Wickham et al., 2019). Covariance among the variables included in the analyses are reported in online Supplemental Table S2.

Our analyses comprised several steps using linear mixed models (LMM). Random effects included subject ID and study site. Right or left hippocampal volume were the dependent variables and fixed effects included Time (age), total intracranial volume, FreeSurfer´s Euler number, sex, socioeconomic status, and the presence of any DSM-IV disorder according to DAWBA´s interview clinical ratings. Right and left hippocampal volumes were investigated in separated models. Given that previous literature found non-linear patterns of cortical and subcortical neurodevelopment, (Bethlehem et al., 2022; Uematsu et al., 2012) we first tested the model-fit parameters for models including linear *v*. quadratic associations of age with hippocampal volume. Akaike Information Criterion (AIC) was used to compare the models and to determine which had the best fit.

Second, to investigate the effect of CM on developmental trajectories of hippocampal volume, we included CM variable (high *v.* low exposure groups) to the previous models. Then, we tested relevant moderation effects by adding to separate models the following interaction terms: i. We tested whether there was a significant Time-by-CM interaction, suggesting longitudinal effects of CM on hippocampal development; ii. We investigated whether there was a moderation of CM by any Mental Disorder and; iii. We explored whether there were varying effects between high and low exposure to maltreatment on hippocampus volume according to sex by including a Sex-by-CM interaction term. Third, we included MDD to the models to investigate whether the previous association between CM and hippocampal development was altered after adjusting for MDD.

Finally, we performed three sensitivity analyses. First, we explored CM as a dimensional variable, using individual factor scores from the CFA on CM items, which merged both parent- and self-reported data. Second, we further explored whether hippocampal volume PRS could have additional effect on the relationship between maltreatment and hippocampal volume by incorporating PRS and the first 10 PCs as covariates in CM models. Third, as an additional strategy to improve the robustness of the trajectory estimations, we performed analyses only including individuals who had at least 2 brain scans.

## Results

### Demographics and clinical characteristics

Demographics and clinical features of the participants are reported in Table 1. At baseline assessment, 42.9% of the sample were female and the mean age was 9.9 years (s.d. = 1.8 year). The groups with high and low exposure to CM did not show significant differences in age, sex, socioeconomic scores and ethnicity (online Supplementary Table S3). The prevalence of psychiatric disorders at each phase of data collection according to the DSM-IV criteria are reported in online Supplementary Table S4. The association between any mental disorder and CM exposure level is depicted in online Supplementary Table S5. Briefly, CM high level group displayed higher rates of any psychiatric disorder as compared to the Low CM group at cohort baseline.

### Hippocampal volumetric development

#### Relationship between childhood maltreatment and hippocampal volume

We used mixed model analyses to investigate the effect of CM (high *v.* low exposure) on right and left hippocampal volume separately. First, we observed that, in a model including all covariates but CM, the quadratic term of age (i.e. age^2^) presented a better fit to the data (right hippocampus model AIC = 2739.71; left hippocampus model AIC = 2760.19) when compared to the model that only included the linear age term (i.e. age) (right hippocampus model AIC = 2753.49; left hippocampus model AIC = 2767.50). The quadratic term of age was significantly associated with both right (*F* = 15.75 *β* = −0.01, *t* = −3.04, *p* = <0.001) and left (*F* = 9.23 *β* = −0.01, *t* = −3.97, *p* = <0.001) hippocampus, showing a longitudinal change of hippocampal volumes according to age in an inverted *U* shape way. Consequently, all further models included the quadratic effect of age.

**Figure 1.**
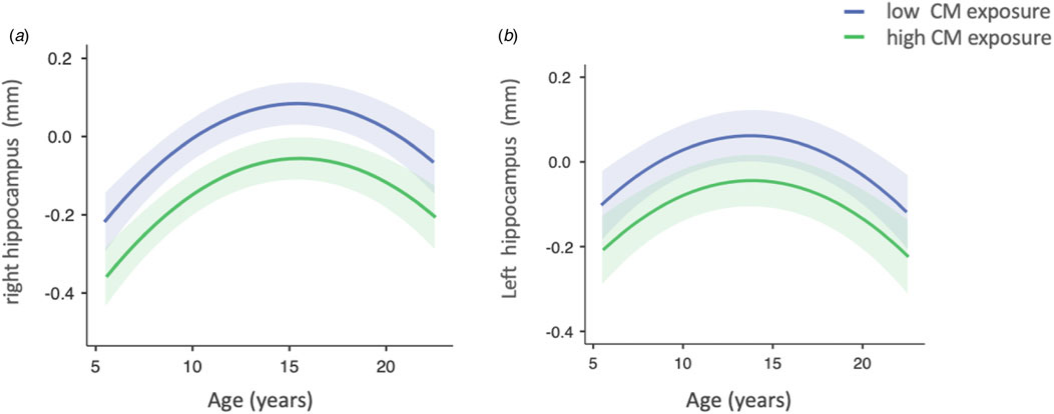
Implied mediation model underlying the reciprocal associations between social processes and psychopathology, and the effects of risk/protective factors.

Second, we found that CM was significantly associated with the volume of the right hippocampus (*F* 4.88 = *β* = −0.14, t = −2.21, *p* = 0.027) (Table 2). As depicted in Fig. 1, reduced right hippocampal volume was associated with higher CM exposure. CM variable did not show a significant main effect on left hippocampal volume (*F* = 2.83, *β* = −0.11, *t* = −1.7, *p* = 0.093) (Table 2). Then, we tested for moderation effects and there were no significant interactions for: i. Time-by-CM (right; *F* = 0.40, *β* = −0.01, *t* = −0.63, *p* = 0.524; left hippocampus *F* = 0.30, *β* = 0.01, *t* = 0.56, *p* = 0.576); ii. Any mental disorder-by-CM (right :*F* = 2.76, *β* = −0.10, *t* = 1.65, *p* = 0.097; left hippocampus *F* = 0.13, *β* = 0.01, *t* = 0.38, *p* = 0.704); or sex-by-CM (right, *F* = 0.59, *β* = −0.10, *t* = 0.77, *p* = 0.443; left hippocampus *F* = 0.41, *β* = 0.07, *t* = 0.65, *p* = 0.517) (online Supplemental Table S6).

**Table 2.** Mixed model analyses to investigate the effect of child maltreatment (high v. low exposure) on hippocampal volume

Child maltreatment				
	*β*	s.e.	*T* test	*p* Value
Right hippocampus				
Child maltreatment	−0.14	0.05	−2.21	**0.027**
Time	0.10	0.01	4.30	**<0.001**
Time(age)^2^	−0.00	0.06	−3.94	**<0.001**
Sex	−0.10	0.05	−1.68	0.094
Left hippocampus				
Child maltreatment	−0.11	0.05	−1.67	0.093
Time	0.07	0.03	2.95	**0.003**
Time (age)^2^	−0.00	0.00	−3.03	**0.003**
Sex	−0.05	0.06	−0.82	0.415
Child maltreatment + major depressive disorder				
	*β*	s.e.	*T* test	*p* Value
Right hippocampus				
Childhood maltreatment	−0.14	0.05	−2.19	**0.029**
Major depression disorder	−0.08	0.05	−1.54	0.121
Time	0.10	0.01	4.31	**<0.001**
Time(age)^2^	−0.00	0.00	−3.89	**<0.001**
Sex	−0.10	0.05	−1.60	0.109
Left hippocampus				
Childhood maltreatment	−0.10	0.05	−1.65	0.099
Major depression disorder	−0.10	0.04	−2.20	**0.027**
Time	0.07	0.01	2.93	**0.003**
Time(age)^2^	−0.00	0.00	−2.94	**0.003**
Sex	−0.03	0.06	−0.70	0.478

*Note:* All models were adjusted for socioeconomic score, any mental disorder, total intracranial volume and FreeSurfer´s Euler number for each time point. Number of observations: 1525, participants included: 795. Random components: ID and study site.

Third, we included MDD to the previous models. The main effect of CM on right hippocampal volume remained significant after including MDD as a predictor in the model (*F* = 4.77, *β* = −0.14, *t* = −2.19, *p* = 0.029). While MDD was not significantly associated with right hippocampus volume (*F* = 2.41, *β* = −0.08, *t* = −1.7X *p* = 0.121), it was associated with lower left hippocampal volume (*F* = 4.88, *β* = −0.11, *t* = −2.21, *p* = 0.027) (Table 2).

### Sensitivity analyses

We performed three sensitivity analyses. First, we tested the association of CM – as measured by a dimensional variable representing individual CM factor scores – with hippocampal development. In the same direction as the categorical CM variable, the dimensional measure of CM presented a statistically significant main effect for right hippocampus (*F* = 7.61, *β* = −0.15, *t* = −2.76, *p* = 0.006), while this association was not significant for the left hippocampus model (*F* = 3.82, *β* = −0.10, *t* = −1.94, *p* = 0.051) (Table 3). Second, to explore whether the associations between CM and hippocampal volume remained consistent after adjusting for potential genetic determinants of hippocampal volume, we re-ran our main models adjusting for hippocampal PRS. As shown in Table 3, we still observed a significant main effect of CM on the right hippocampus (*F* = 4.20, *β* = −0.13, *t* = −2.04, *p* = 0.041). There was no significant main effect of CM on left hippocampal volume (*F* = 2.33, *β* = −0.10, *t* = −1.53, *p* = 0.128). Third, we performed sensitivity analyses by including individuals with at least two brain scans. There was a significant effects for right hippocampal volume (*F* = 5.19, *β* = −0.16, *t* = −2.27, *p* = 0.023) (online Supplementary Table S7) and no significant effect for the left hippocampus (*F* = 1.40 *β* = −0.08, *t* = −1.19, *p* = 0.237).

**Table 3.** Sensitivity analyses: mixed model to investigate the effect of child maltreatment (high v. low exposure) on hippocampal volume i. Using a dimensional measure of child maltreatment and ii. Adjusting for hippocampal volume polygenic risk score

Child maltreatment as a dimensional variable				
	*β*	s.e.	*T* test	*p* Value
Right hippocampus^a^				
Child maltreatment dimensional	−015	0.04	−2.76	**0.006**
Time	0.10	0.01	4.38	**< 0.001**
Time (age)^2^	−0.00	0.00	−4.00	**< 0.001**
Sex	−0.10	0.05	−1.70	0.088
Left hippocampus^a^				
Child maltreatment dimensional	−0.10	0.04	−1.94	0.051
Time	0.07	0.01	2.10	**0.003**
Time (age)^2^	−0.00	0.00	−3.07	**0.002**
Sex	−0.05	0.06	−0.82	0.405
Child maltreatment + polygenic risk score for hippocampal volume				
	*β*	s.e.	*T* test	*P* Value
Right hippocampus^b^				
Child maltreatment	−0.133	0.07	−2.04	**0.041**
Time	0.10	0.01	4.22	**< 0.001**
Time(age)^2^	−0.00	0.00	−3.81	**< 0.001**
Sex	−0.10	0.05	−1.62	0.107
PRS	0.00	0.00	1.10	0.179
Left hippocampus^b^				
Child maltreatment	−0.10	0.05	−1.53	0.128
Time	0.06	0.01	3.26	**0.001**
Time(age)^2^	−0.00	0.00	−3.27	**0.001**
Sex	−0.05	0.05	−0.82	0.413
PRS	0.00	0.00	1.19	0.236

PRS, polygenic risk score of hippocampus.

a Number of observations: 1 525, participants included: 795.

b Number of observations: 1269, participants included: 539.

*Note:* All models were adjusted for socioeconomic score, any mental disorder, total intracranial volume, and FreeSurfer´s Euler number for each time point.

## Discussion

We analyzed longitudinal data to investigate hippocampal volumetric trajectories across childhood and adolescence among individuals with a history of CM. We observed reduced right hippocampal volume at baseline in individuals who were exposed to high levels of CM and showed that this effect was consistent over time. Therefore, our findings suggest that CM impacts right hippocampal volume in a sustained fashion from early to late adolescence. Further, we demonstrated the robustness of this main finding as it persisted in several follow-up analyses, including those that adjusted for the effect of psychopathology, namely MDD, and for the influence of some genetic determinants on hippocampal volume.

Structural volumetric reductions in the right hippocampus observed here are consistent with prior CM studies in children and adolescents (Hanson et al., 2015; Paquola et al., 2017; Whittle et al., 2017). While we identified a significant quadratic effect of age on hippocampal growth, we did not observe a significant CM-moderated change in hippocampal development over time, given that the time-by-CM interaction terms were not statistically significant. This finding contrasts with a previous study showing that participants reporting CM exhibited altered hippocampal volume during early adolescence but then presented a volumetric ‘catch-up’ with the ‘normative’ non-maltreated group by late adolescence (Whittle et al., 2017). Differences in sample size and sex distribution between these studies might explain the discrepancy. In accordance with previous literature, we found no evidence of a significant sex-by-CM interaction in hippocampal volume (Paquola et al., 2017).

We will now address some potential mechanisms underlying the CM-hippocampal volumetric link found in the present study. CM has been associated with dysregulation of the hypothalamic–pituitary–adrenal (HPA) axis (Gunnar & Quevedo, 2007), initiating a cascade of stress-mediated hormonal activation that leads to an elevation of glucocorticoid hormones, stress-induced neurotransmitters, and inflammatory cytokines. Thus, stress targets hippocampal structure via glucocorticoid receptor activation, and can induce various cellular and systemic effects on synaptic plasticity, neuronal survival, hippocampal neurogenesis and connectivity (Bartsch & Wulff, 2015; McEwen, Nasca, & Gray, 2016). This particular vulnerability is related to hippocampal high degree of plasticity, high glucocorticoid receptor density, protracted ontogeny, and persistent postnatal neurogenesis (McEwen et al., 2016).

Consistent with a previous longitudinal study, we did not observe a relationship between CM and left hippocampal volume neurodevelopment (Paquola et al., 2017). Interestingly, a recent study has shown that CM indirectly predicted a decrease in left hippocampal development, while the co-morbidity with psychopathology significantly mediated the relationship between CM and reduced left hippocampus (Whittle et al., 2013). Distinct developmental peaks (Uematsu et al., 2012) or functional specialization (Iglói, Doeller, Berthoz, Rondi-Reig, & Burgess, 2010) may explain why the right hippocampus is more sensitive to CM compared to the left hippocampus. Our findings suggest that continued assessment of right *v.* left brain differences in vulnerability (or sensitive periods) to environmental influences during neurodevelopment is warranted (Lupien, McEwen, Gunnar, & Heim, 2009).

Prior studies of adolescents with MDD have found reduced volume of the hippocampus (Jaworska et al., 2016; Straub et al., 2019). Moreover, previous literature has consistently implicated CM as a risk factor for youth MDD (Jaffee, 2017). Critically, we found that the effect of CM on hippocampal development was observed even after adjusting for the presence of MDD. This finding suggests that the effect of CM on right hippocampus volumes occurred beyond the potential deleterious impacts of MDD to the hippocampal formation. It is important to note that the relationship between maltreatment, depression, and hippocampal volume may be complex and multifaceted, and there may be other unconsidered factors influencing these associations.

Our study assessed maltreatment only at baseline, which may consider ‘early child maltreatment’. A primary limitation of having conducted a single assessment of childhood maltreatment in early childhood is that it may not have fully captured the entirety of the maltreatment experience over time. Childhood maltreatment is a complex phenomenon that can vary in its frequency, severity, and nature throughout a child's development. By conducting only one assessment at the outset, there is a risk of underestimating subsequent maltreatment prevalence. Even so, for the present analyses, we reported a relatively high frequency of CM among our population. We employed a previously published data-driven method to classify participants into High *v.* Low exposure to CM (Salum et al., 2016). This method avoids imposing pre-determined thresholds for CM, such as arbitrarily deciding which frequency or intensity determine a positive High *v.* Low rating for exposure. Moreover, prevalence of CM may differ significantly across regions, reflecting specific socio-cultural contexts or methodologies employed in research (Stoltenborgh, Bakermans-Kranenburg, Alink, & van IJzendoorn, 2015). For instance, official reports by child protective service agencies in the United States estimate that approximately 12.5% of children will experience CM by the age of 18 (Wildeman et al., 2014). In contrast, studies conducted in South America have highlighted notably higher rates of maltreatment, such as prevalence rates of 19.7% for self-reported emotional neglect (Soares et al., 2016). Therefore, CM prevalence rates reported in the present study may aligns with estimates previously seen other developing countries.

### Limitations

The present study has several strengths, such as, a three-wave longitudinal design, a relatively large sample size, and a community-based sample from an underrepresented population. There are of course limitations that must be addressed. The CM measures implemented in the present study must first be considered. First, our CM assessment relied on self- and parent-report and, thus, may be prone to recall bias. Optimally, these data should be aggregated with other sources of information (e.g. other family members, child protective services, health and educational systems) or observational research protocols of the family environment (Melhuish, Belsky, Leyland, Barnes, & Team, 2008). A strength of our assessment is that interviews were performed by different interviewers in diverse locations (school-based interview for children and household interview for parents).

Second, previous studies have reported low agreement between parent–child reports for CM (Devries et al., 2018; Kobulsky, Kepple, Holmes, & Hussey, 2017). Indeed, child-reported CM is typically lower than that reported by the main caregiver (Devries et al., 2018). To address this issue, we aggregated both child- and parent-reports of CM. Further, we invoked a data-driven approach to determine high and low CM exposure groups, which diminished potential bias introduced by arbitrary researcher-based definitions for what constitutes high-level CM. Moreover, we note that both categorical and dimensional data-derived CM measures were associated with altered right hippocampal volumes.

Third, our CM measure comprised different subtypes of CM. Given our sample size, we did not think it prudent to analyze the data according to the different subtypes of CM. Though data suggest that children who experience one kind of victimization are at an increased risk of experiencing other forms of victimization (Finkelhor, Ormrod, & Turner, 2007), distinct CM experiences may impact hippocampal development in different ways (Herzog et al., 2020). In a related point, children exposed to CM are at an increased risk of recurrent exposure (Connell, Bergeron, Katz, Saunders, & Tebes, 2007), which in turn may decrease potential developmental ‘catch-up’ effects. Thus, the investigation of specific subtypes of CM as well as the effects of repeated exposure to maltreatment, and their effect on hippocampal development, remain important questions for future research. Fourth, the effects of artifacts, such as head movement, may introduce biases in the estimation of brain volumes in neuroimaging research (Ducharme et al., 2016). Even though we did not perform visual quality control check for hippocampal segmentations for all participants, we revised all outliers and excluded cases with notable segmentation errors. Moreover, we have included FreeSurfer´ Euler number variable in all statistical models, which is a quality control measure to enhance the accuracy of potential individual segmentation errors. Finally, we did not correct for multiple comparisons, such as those involving separated analyses of the left and right hippocampus or the number of tested models. On the other hand, our main finding remained robust to the scrutiny of several sensitivity analyses.

## Conclusions

The present finding of sustained reduction of right hippocampal volume across childhood and adolescence in individuals who were exposed to high levels of CM, provides novel insight into the neurodevelopmental susceptibility to deleterious environmental factors. If replicated, this finding underscores the urgent need for interventions that minimize – or even remedy – the impact of early stressors, such as CM, on the brain.

## Supporting information

Doretto et al. supplementary material Doretto et al. supplementary material

## Supplementary material

The supplementary material for this article can be found at https://doi.org/10.1017/S0033291724001636
