# Supplementary material for: Childhood maltreatment and the structural development of hippocampus across childhood and adolescence
Source: Psychol Med. 2025 Jan 8;54(16):4528–36. doi: 10.1017/S0033291724001636 (PMC11769901; doi:10.1017/S0033291724001636)
Supplement: Doretto et al. supplementary material [file S0033291724001636sup001.docx]

# Supplementary Material

## *Supplementary Methodology*

## *Participants*

The final sample composition of the study sample is shown in Figure S1.

## *Table S1.* Flowchart of sample composition of the study

### Childhood Maltreatment Assessment

Responses to seven questions were rated on a 4-point scale: 0, never; 1, one or two times; 2, sometimes; 3, frequently. Trained lay interviewers administered the research protocol to parents, using the following questions:

- Has [name of the child] ever been seriously beaten by an adult (including yourself) at home, hurting him/her or leaving bruises or marks?

- Has [name of the child] ever not had enough to eat or been forced to use dirty or torn clothes? - Has [name of the child] ever been cursed with words like stupid, idiot, dumb or useless or been exposed to someone shouting or screaming?

- Has anyone ever done anything sexual with [name of the child] or threatened to hurt him/her if [name of the child] refused to do it?

Trained clinical psychologists asked children three questions related to Childhood Maltreatment:

- Have you ever been seriously beaten by an adult at home, hurting you or leaving bruises or marks?

- Have you ever not had enough to eat or been forced to use dirty or torn clothes?

- Have you ever been cursed with words like stupid, idiot, dumb or useless or been exposed to someone shouting or screaming?

Dichotomous categorical classification used CFA item-level thresholds:

Participants were classified as “high” exposure to CM if there were:

Parent-report: a) sometimes or frequently exposed to physical abuse or b) sometimes or frequently exposed to physical neglect, c) rarely, sometimes or frequently exposed to sexual abuse, and d) frequently exposed to emotional abuse.

Self-report: a) sometimes or frequently exposed to physical abuse, b) rarely, sometimes or frequently exposed to physical neglect or c) frequently exposed to emotional abuse.

Table S1 – Prevalence of subtypes of early Childhood Maltreatment (as reported at baseline)

|  | **Baseline**  **(717)** |  |  |  | |
| --- | --- | --- | --- | --- | --- |
| **Parents Interview** | (n/%) |  | **Child Interview** |  | |
| **physical abuse** | * |  | | |  |
| 0 | 606 (84.6%) | 603 (84.1%) | | |  |
| 1 | 86 (12.0%) | 66 (9.2%) | | |  |
| 2 | 22 (3.1%) | 42 (5.9%) | | |  |
| 3 | 2 (0.3%) | 6 (0.8%) | | |  |
| **Physical neglect** |  |  | | |  |
| 0 | 641 (89.4%) | 675 (94.1%) | | |  |
| 1 | 54 (7.5%) | 28 (3.9%) | | |  |
| 2 | 18 (2.5%) | 12 (1.7%) | | |  |
| 3 | 4 (0.6%) | 2 (0.3%) | | |  |
| **Emotional abuse** |  |  | | |  |
| 0 | 385 (52.7%) | 550.0 (76.7%) | | |  |
| 1 | 134 (18.7%) | 76 (10.6%) | | |  |
| 2 | 157 (21.9%) | 67 (9.3%) | | |  |
| 3 | 48 (6.7%) | 24 (3.3%) | | |  |
| **Sexual abuse** ** |  |  | | |  |
| 0 | 794 (97.2%) |  | | |  |
| 1 | 19 (2.7%) |  | | |  |
| 3 | 1. (0.1%) |  | | |  |

*Note*: 0, never; 1, one or two times; 2, sometimes; 3, frequently. * 1 missing data; ** 3 missing data

***Hippocampus volume across sample***

Figure S2 - Hippocampal volume of right and left hippocampus depicted as a function of age.

A. B.
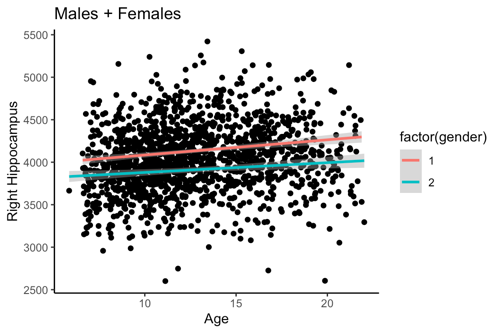

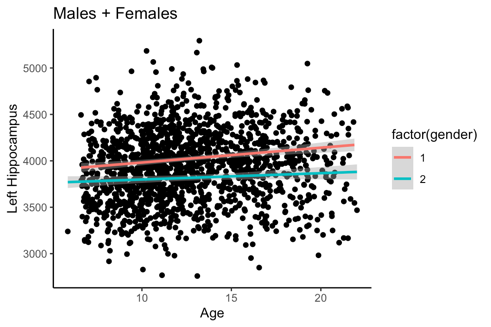


Figure S2. Scatter plots and regression lines depicting the relationship between right (A) and left (B) hippocampus volume and age across the entire sample. Volume in mm2 (y-axis) by age (x-axis). Regression lines for males (red) and females (blue) are presented separately.

Figure S3. Individual trajectories of right and left hippocampal volume depicted as a function of age.

1. B.


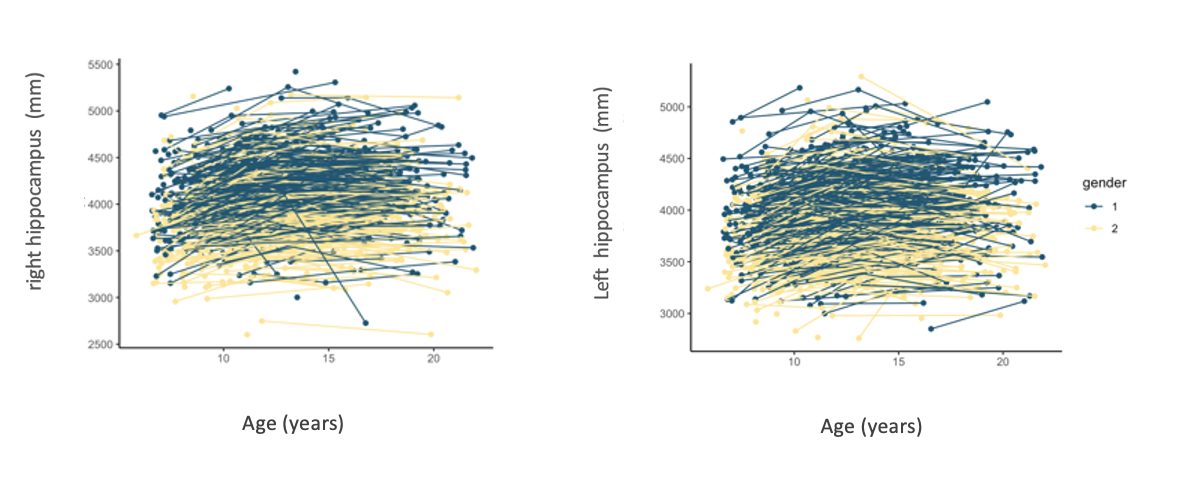


Figure S3 shows individual participants´ trajectories for right (A) and left hippocampus (B) volume across age. Blue lines represent male and yellow lines females. Volume in mm2 (y-axis) by age in years (x-axis) is shown for participants.

Tables S2 - Correlations among study´s variables

| R de Pearson; P value | CM | CM dimensional | Age | Gender | Socioeconomic score | Right Hippocampus | Left Hippocampus | ICV | Euler number | Mental Disorder | Depression | PRS |
| --- | --- | --- | --- | --- | --- | --- | --- | --- | --- | --- | --- | --- |
| CM | - |  |  |  |  |  |  |  |  |  |  |  |
| CM dimensional | 0.64 < .001 |  |  |  |  |  |  |  |  |  |  |  |
| Age | -0.05 0.219 | 0.10 0.005 |  |  |  |  |  |  |  |  |  |  |
| Gender | -0.01 0.848 | 0.00 0.876 | 0.00 0.978 |  |  |  |  |  |  |  |  |  |
| Socioeconomic score | - 0.08 0.040 | -0.13 < .001 | 0.03 0.251 | -0.03 0.473 |  |  |  |  |  |  |  |  |
| Right Hippocampus | -0.09 0.018 | -0.07 0.025 | 0.16 < .001 | -0.27 < .001 | 0.07 0.073 |  |  |  |  |  |  |  |
| Left Hippocampus | -0.05 0.189 | -0.05 0.221 | 0.16 < .001 | -0.24 < .001 | 0.06 0.122 | 0.81 < .001 |  |  |  |  |  |  |
| ICV | -0.04 0.327 | -0.02 0.715 | 0.21 < .001 | -0.42 < .001 | 0.05 0.237 | 0.60 < .001 | 0.60 < .001 |  |  |  |  |  |
| Euler number | 0.02 0.373 | 0.06 0.045 | 0.18 < .001 | 0.10 0.006 | 0.00 0.962 | 0.02 0.637 | 0.04 0.163 | 0.03 0.255 |  |  |  |  |
| Mental Disorder | 0.12 < .001 | 0.26 < .001 | 0.02 0.679 | -0.03 0.441 | -0.06 0.131 | 0.04 0.343 | 0.03 0.460 | -0.00 0.857 | -0.07 0.024 |  |  |  |
| Depression | 0.11 < .001 | 0.22 < .001 | 0.06 0.135 | 0.08 0.034 | -0.04 0.332 | -0.01 0.728 | - 0.02 0.400 | -0.00 0.848 | -0.02 0.417 | 0.30 < .001 |  |  |
| PRS | 0.03 0.449 | -0.00 0.802 | 0.03 0.452 | -0.03 0.278 | 0.04 0.317 | 0.05 0.201 | 0.05 0.099 | 0.03 0.253 | -0.05 0.089 | 0.00 0.870 | -0.00 0.968 | - |

*Note:* CM, Childhood Maltreatment; ICV, Intracranial Volume; PRS, Polygenic Risk Score.

***Supplementary Results***

Table S3 - Demographics and Clinical Characteristics of Participants according to Childhood Maltreatment group

|  | **Childhood maltreatment** | | | | **p-value** | | | |  | | |  |
| --- | --- | --- | --- | --- | --- | --- | --- | --- | --- | --- | --- | --- |
|  | **High exposure** | | **Low exposure** | |  | | | | **Total = 717** | | |  |
| subjects, n |  |  |  | | |  |  | | |  |  |  |
| W0 | 163 |  | 554 | | |  |  | | |  | | |
| W1 | 111 |  | 323 |  | |  | |  | | |  |  |
| W2 | 93 |  | 281 |  | |  | |  | | |  |  |
|  |  |  |  |  | |  | |  | | |  |  |
| Age (years), mean (SD) |  |  |  |  | |  | |  | | |  |  |
| W0 | 9.8 (1.9) |  | 10.0 (1.8) |  | | 0.066^1^ | |  | | |  |  |
| W1 | 12.9 (1.9) |  | 13.0 (1.8) |  | | 0.446^1^ | |  | | |  |  |
| W2 | 17.7(2.1) |  | 17.6 (2.8) |  | | 0.514 ^1^ | |  | | |  |  |
|  |  |  |  |  | |  | |  | | |  |  |
| **Sociodemographic at baseline** | | | | | | | | | | |  |  |
|  |  |  |  |  | |  | |  | | |  |  |
| Female, (%) | 41.7 |  | 43.0 |  | | 0.778^2^ | |  | | |  |  |
| Site, (%) |  |  |  |  | |  | |  | | |  |  |
| Sao Paulo | 47.2 |  | 51.4 |  | | 0.345^2^ | |  | | |  |  |
|  |  |  |  |  | |  | |  | | |  |  |
| Socioeconomic score, mean (SD) | 17.6 (4.3) |  | 18.3 (4.45) |  | | 0.051^1^ | |  | | |  |  |
|  |  |  |  |  | |  | |  | | |  |  |

*Note:* n, number; SD, standard deviation . ^1^ = Pearson ; ^2^ = Chi-square test.

Table S4 –Prevalence of psychiatric disorders among participants according to the DSM-4 criteria at each phase of data collection

| **Psychiatric disorder DSM-IV** | **Wave 0 (N=717)** | **Wave 1 (N=434)** | **Wave 2 (N=374)** | **Total**  **(N=1525)** |
| --- | --- | --- | --- | --- |
| Any psychiatric disorder | 221.0 (30.8%) | 157.0 (36.2%) | 114.0 (30.5%) | 492.0 (32.3%) |
| Post- traumatic stress disorder | 10.0 (1.4%) | 6.0 (1.4%) | 7.0 (1.9%) | 23.0 (1.5%) |
| Any ADHD/hyperactive disorder | 87.0 (12.1%) | 36.0 (8.3%) | 17.0 (4.5%) | 140.0 (9.2%) |
| Any conduct/oppositional disorder | 52.0 (7.3%) | 38.0 (8.8%) | 11.0 (2.9%) | 101.0 (6.6%) |
| Any anxiety disorder * | 54.0 (7.5%) | 58.0 (13.4%) | 51.0 (13.6%) | 163.0 (10.7%) |
| Any depression | 28.0 (3.9%) | 49.0 (11.3%) | 60.0 (16.0%) | 137.0 (9.0%) |

*Note:* *Panic Disorder, Agoraphobia, Generalized Anxiety Disorder, Social Phobia and Specific Phobia

Table S5 - Prevalence of mental disorders between groups with high and low levels of Childhood Maltreatment at cohort baseline

| **Psychiatric disorder DSM-IV** | **Low MT (N=554)** | **High MT**  **(N=163)** | **Total**  **(N=717)** | **p value**^1^ |
| --- | --- | --- | --- | --- |
| Any psychiatric disorder | 153.0 (27.6%) | 68.0 (41.7%) | 221.0 (30.8%) | < 0.001^1^ |
| Post- traumatic stress disorder | 3.0 (0.5%) | 7.0 (4.3%) | 10.0 (1.4%) | < 0.001^1^ |
| Any ADHD/ hyperactive disorder | 64.0 (11.6%) | 23.0 (14.1%) | 87.0 (12.1%) | 0.379^1^ |
| Any conduct/oppositional | 28.0 (5.1%) | 24.0 (14.7%) | 52.0 (7.3%) | < 0.001^1^ |
| Any anxiety disorder * | 38.0 (6.9%) | 16.0 (9.8%) | 54.0 (7.5%) | 0.209^1^ |
| Any depression | 15.0 (2.7%) | 13.0 (8.0%) | 28.0 (3.9%) | 0.002^1^ |

Note: ^1^  Chi-square test. *Panic Disorder, Agoraphobia, Generalized Anxiety Disorder, Social Phobia and Specific Phobia

Table S6 - Mixed model analyses to investigate the effect of Child Maltreatment (high vs low exposure) on hippocampal volume when relevant interactions were included in the models.

| **Child Maltreatment by time interaction** | | | | | |
| --- | --- | --- | --- | --- | --- |
| **Right Hippocampus** |  |  |  |  |  |
| Child Maltreatment | -0.36 | 0.30 | -1.22 | 0.223 |  |
| Time | 0.10 | 0.01 | 4.23 | **< .001** | |
| Time(age) ² | -0.00 | 0.00 | -3.90 | **< .001** | |
| CM * Time(age) ² | -0.00 | 0.00 | -0.63 | 0.524 |  |
| sex | -0.10 | 0.06 | 0.10 | 0.092 |  |
| **Left Hippocampus** |  |  |  |  |  |
| Child Maltreatment | 0.11 | 0.30 | 0.34 | 0.723 |  |
| Time | 0.06 | 0.02 | 2.42 | **0.015** | |
| Time(age) ² | -0.00 | 0.00 | -2.54 | **0.011** | |
| CM * Time(age) | -0.03 | 0.03 | -0.64 | 0.514 |  |
| CM * Time(age) ² | 0.00 | 0.00 | 0.56 | 0.576 |  |
| sex | -0.05 | 0.06 | -0.80 | 0.422 |  |
| **Child Maltreatment by Any Mental Disorder interaction** | | | | | |
| **Right Hippocampus** |  |  |  |  |  |
| Child Maltreatment | -0.129 | 0.06 | -2.03 | **0.043** |  |
| Time | 0.10 | 0.01 | 4.36 | **< .001** | |
| Time(age) ² | -0.00 | 0.00 | -3.98 | **< .001** | |
| sex | -0.10 | 0.06 | -1.72 | 0.086 |  |
| CM * any mental disorder | 0.10 | 0.05 | 1.65 | 0.097 |  |
| **Left Hippocampus** |  |  |  |  |  |
| Child Maltreatment | -0.10 | 0.05 | -1.62 | 0.103 |  |
| Time | 0.066 | 0.01 | 2.94 | **0.003** | |
| Time(age) ² | -0.00 | 0.00 | -3.03 | **0.002** | |
| sex | -0.05 | 0.06 | -0.81 | 0.411 |  |
| CM * any mental disorder | 0.01 | 0.05 | 0.38 | 0.704 |  |
| **Child Maltreatment by sex interaction** | | | | | |
| **Right Hippocampus** |  |  |  |  |  |
| Child Maltreatment | -0.12 | 0.05 | -2.05 | **0.040** |  |
| Time | 0.08 | 0.01 | 4.30 | **< .001** | |
| Time(age) ² | -0.00 | 0.00 | -3.95 | **< .001** | |
| sex | -0.08 | 0.07 | -1.10 | 0.271 |  |
| CM * sex | 0.10 | 0.13 | 0.77 | 0.443 |  |
| **Left Hippocampus** |  |  |  |  |  |
| Child Maltreatment | -0.10 | 0.05 | -1.56 | 0.119 |  |
| Time | 0.07 | 0.01 | 2.94 | **0.003** | |
| Time(age) ² | -0.00 | 0.00 | -3.02 | **0.002** | |
| sex | -0.03 | 0.07 | -0.40 | 0.687 |  |
| CM * sex | 0.07 | 0.13 | 0.65 | 0.517 |  |

*Note:* The model was adjusted for socioeconomic score, mental disorder, intracranial volume, euler’s number for each time point. CM, Childhood Maltreatment. Number of Observations: 1525; Participants Included n= 795.

***Sensitivity Analyses***

We re-ran our main models including individuals who had at least 2 brain scans as an additional strategy to improve the robustness of the hippocampal developmental trajectories´ estimations.

Table S7. Models including individuals who had at least 2 brain scans

| **Characteristic** | **β** | **SE** | **T-test** | **P Value** |  |
| --- | --- | --- | --- | --- | --- |
| **Model 1** |  |  |  |  |  |
| **Right Hippocampus** |  |  |  |  |  |
|  |  |  |  |  |  |
| Child Maltreatment | -0.16 | 0.08 | -2.27 | **0.023** |  |
| Time | 0.10 | 0.01 | 4.50 | **< .001** | |
| Time(age) ² | -0.00 | 0.00 | -4.08 | **< .001** | |
| sex | -0.08 | 0.06 | -1.30 | 0.190 | |
| **Left Hippocampus** |  |  |  |  |  |
| Child Maltreatment | -0.09 | 0.06 | -1.19 | 0.237 |  |
| Time | 0.06 | 0.01 | 3.22 | **0.001** | |
| Time(age) ² | -0.00 | 0.00 | -3.18 | **0.002** | |
| sex | -0.02 | 0.06 | -0.24 | 0.801 |  |

*Note:* The model was adjusted for socioeconomic score, mental disorder, intracranial volume, euler’s number for each time point. CM, Childhood Maltreatment. Number of Observations: 1269; Participants Included n: 539.
